# Structural Basis for how Sialoglycan-binding Viridans Streptococci Accommodate Ligands that Exceed the Characterized Binding Site

**DOI:** 10.17912/micropub.biology.001838

**Published:** 2026-01-02

**Authors:** KeAndreya M Morrison, Kole Martin, Hai Yu, Xi Chen, TM Iverson

**Affiliations:** 1 Department of Pharmacology, Meharry Medical College, Nashville, TN, US; 2 Department of Pharmacology, Vanderbilt University, Nashville, TN, US; 3 Department of Chemistry, UC Davis, Davis, CA, US; 4 Department of Biochemistry, Vanderbilt University, Nashville, TN, US

## Abstract

During endocardial infections, viridans group streptococci use proteins containing siglec-like binding regions to engage sialic acid-capped *O-* GalNAc glycans on platelet glycoprotein GPIbα. Much past work used isolated di-, tri-, or tetrasaccharide partial ligands to interrogate this sialoglycan binding. Here, we report the 1.9 Å resolution crystal structure of the *Streptococcus gordonii* strain M99 siglec-like binding region bound to an L-serine-linked sialyl T antigen (sTa) trisaccharide. The structure demonstrates how trisaccharide extensions are accommodated, with implications for binding larger sialoglycan ligands.

**Figure 1. Structure of sTa-( N -Fmoc)Ser bound to SLBR GspB:**
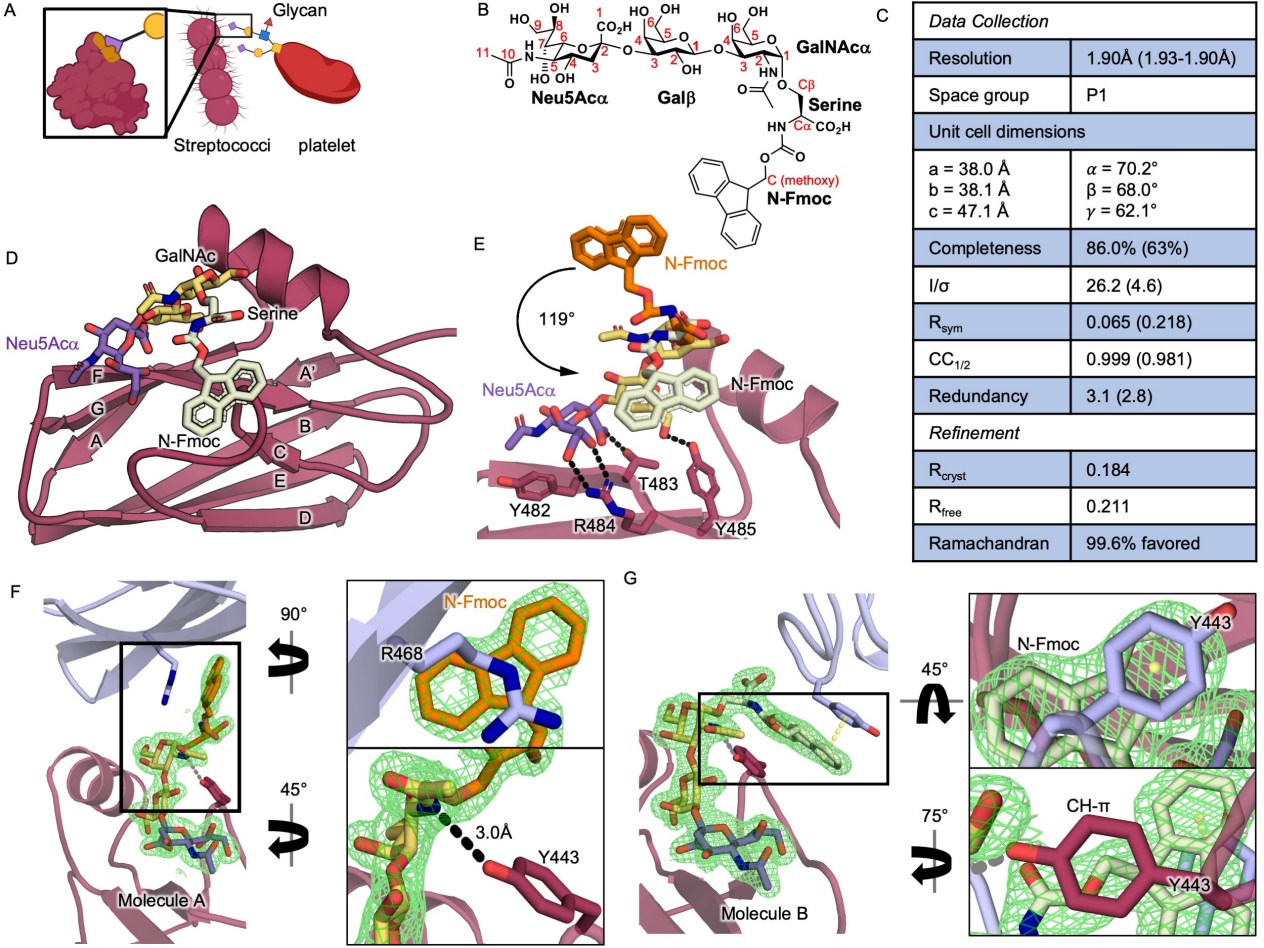
A) Cartoon of streptococci engaging platelet sialoglycans, **B)** sTa-( *N* -Fmoc)Ser, **C)** Crystallographic data collection and refinement statistics. Values in parentheses are for the highest resolution shell, **D)** Ribbons diagram of SLBR _GspB_ with the strands labeled. Glycans are colored according to SNFG convention, **E)** Superposition of sTa-( *N* -Fmoc)Ser-SLBR _GspB_ in molecule A versus molecule B showing ( *N* -Fmoc)Ser is rotated 119° around the O4 bond. Hydrogen bonds are shown in black lines. **F, G)** Close up view of sTa-( *N* -Fmoc)Ser-SLBR _GspB_ from **F)** Molecule A and **G)** Molecule B of the model superimposed with composite omit electron density contoured at 1σ ( *green mesh* ). The insets show: **F)** the interaction between the N-Fmoc cyclopentane of molecule A and R468 side chain from a symmetry-related molecule ( *pale blue* ) or the hydrogen-bond between Tyr443 and GalNAc N2; and **G)** the π stacking quadrupole interaction between a Fmoc benzene group and Tyr443 from molecule A ( *yellow line* ) or the CH-π interaction between the Fmoc methoxy carbon and Tyr443 from molecule B.

## Description

Viridans group streptococci are a major causative agent of bacterial infective endocarditis, a serious infection of the heart valves (Li et al., 2024; Talha et al., 2020). The first committed step of these infections is high affinity binding of streptococci to platelets, which occurs when a siglec-like binding region (SLBR) within a larger adhesive protein engages α2,3-sialylated *O* -GalNAc glycans on platelet glycoprotein GPIbα ( **Figure 1A** ) (Chahal et al., 2022). A binding preference for glycans containing sialyl T antigen (sTa, Neu5Acα2–3Galβ1–3GalNAcα) correlates with virulence in animal models of endocardial infection, prompting interest in SLBR binding repertoire (Bensing et al., 2019).

Past work to assess sialoglycan selectivity frequently leveraged an in vitro reductionist approach that assessed how synthetic di-, tri-, and/or tetrasaccharides bind either SLBRs or a smaller “siglec-like” subdomain. Collectively, published molecular work now spans >10 SLBR homologs and identifies: (a) a ФTRX sequence motif for sialic acid binding; (b) how sialic acid forms are distinguished; (c) unique selectivity repertoires for closely related SLBRs; and (d) how selectivity loops engage distinct glycans. These studies advance our understanding of SLBR-dependent host-pathogen interactions (Bensing et al., 2022; Deng et al., 2014; Di Carluccio et al., 2024; Di Carluccio et al., 2021; Loukachevitch et al., 2016; Morrison et al., 2025; Pyburn et al., 2011; Stubbs et al., 2020).

A recurring theme, however, is that the reductionist system does not always fully recapitulate the larger binding event (Morrison et al., 2025; Takamatsu et al., 2005). It is now appreciated that the short synthetic sialoglycans used in many studies represent only segments of the biological ligand(s) (Bensing et al., 2022; Chahal et al., 2022; Di Carluccio et al., 2021; Loukachevitch et al., 2016). One known feature of *O* -GalNAc glycan-containing biological ligands is attachment via a serine, threonine, or less commonly tyrosine residue on the protein (Varki, 2017). To explore whether a linked amino acid residue, such as a serine, affects sialoglycan binding (Bensing et al., 2019), we chose sTa-( *N* -Fmoc)Ser in which the amino group of the serine is linked to a 9-fluorenylmethyloxycarbonyl (Fmoc) group (Lau et al., 2011) ( **Figure 1B** ). To ensure that any observed binding of this compound is not due to intrinsic SLBR promiscuity, we chose the SLBR from *Streptococcus gordonii* strain M99, termed SLBR _GspB_ , which only bound to sTa in glycan arrays containing 74 sialylated glycans and non-sialylated controls (Bensing et al., 2016).

We crystallized the siglec-like subdomain of SLBR _GspB_ (SLBR _GspB-Siglec_ ) with sTa-( *N* -Fmoc)Ser and determined the 1.9 Å-resolution structure ( **Figure 1C, Figure 1D** ). The crystal contains two mathematically distinct molecules of SLBR _GspB-Siglec_ in each asymmetric unit, molecule A and molecule B. These two molecules have an RMS deviation of 0.48 Å for the Ca atoms. Calculations in PISA (Krissinel and Henrick, 2007) identify a maximal buried interface of 550 Å, which is below the threshold for stable oligomerization. Consistent with past work in solution, this supports the assignment of SLBR _GspB_ as a monomer with two copies in the asymmetric unit, and not a dimer (Pyburn et al, 2011).

We focus our comparative analysis of molecules A and B on the structural features that are likely of biological relevance. For the sTa trisaccharide, superposing molecule A and molecule B gives an RMS deviation of 0.19 Å for all atoms, which is within the coordinate error. The sTa hydrogen-bonding in this sTa-( *N* -Fmoc)Ser-SLBR _GspB_ costructure ( **Figure 1E** ) is also within coordinate error of that observed in the costructure of SLBR _GspB_ with isolated sTa (PDB 5IUC (Pyburn et al., 2011)).

For the GalNAc-α-Ser glycosidic linkage and serine, both are ordered in the two molecules of the asymmetric unit but neither directly contacts SLBR _GspB_ . Serine ordering in the absence of protein contacts likely results from tethering between the tightly bound sTa and the tightly bound Fmoc; in a superposition, the serines are rotated with respect to each other by 41°. As the combined sTa and serine interactions do not differ from those of isolated sTa, which has a micromolar affinity, this argues against sTa-Ser on GPIba being the biological ligand because full ligands exhibit nanomolar affinity and support streptococcal adherence under high shear force in the heart valves (Yakovenko et al., 2018).

The Fmoc components are differently stabilized by crystal contacts, with a 119° rotation around the O4 bond and a maximum displacement of 9.4 Å ( **Figure 1E** ). In molecule A, the Fmoc cyclopentane interacts with the cationic Arg468 side chain from a symmetry-related molecule ( **Figure 1F** ). In molecule B, Fmoc is stabilized by two types of π interactions: (1) Tyr443 from molecule A, which approaches the Fmoc due to crystal packing and forms a quadrupole stacking interaction with one of the benzene components of the Fmoc ring; and more interestingly, (2) the adjacent Tyr443 side chain of the same molecule forms a CH-π interaction with the Fmoc methoxy carbon ( **Figure 1G** ). This latter interaction resembles a glycan binding mode (Kiessling & Diehl, 2021) and suggests how natural ligands might interact with SLBR _GspB_ .

True SLBR ligands are larger *O* -GalNAc-linked and sTa-capped glycans that would exceed the characterized sTa binding site (Bensing et al., 2022; Di Carluccio et al., 2021; Loukachevitch et al., 2016). One possibility for how SLBRs accommodate ligand extensions is through the binding surface occupied by the ordered Fmoc in molecule B, where the CH-π interaction with the Tyr443 could be recapitulated by a glycan. Consistent with this proposal, published studies showed that SLBR _GspB_ with a Y443F mutation has modestly reduced binding to GPIbα glycans (Pyburn et al., 2011), although it is noted that the Tyr443 side chain hydroxyl also forms a long hydrogen-bond to the N2 of GalNAc on sTa (3.0 Å in molecule A or 3.1 Å in molecule B; **Figure 1F- G** ).

Precisely identifying the full ligand and binding site in SLBR _GspB_ and the larger SLBR family will likely require technical advances in the field of glycobiology; complex carbohydrates and glycoconjugates are challenging to be sequenced and synthesized, limiting in vitro work. In the interim, tolerance of sTa-( *N* -Fmoc)Ser by SLBR _GspB-Siglec_ shows how SLBRs accommodate a ligand that exceeds the characterized sTa-binding site, which offers insight into binding larger sTa-containing glycans and improves the understanding of how viridans group streptococci engage glycosylations.

## Methods

*Protein expression and purification.* SLBR _GspB-Siglec_ was expressed and purified as described, using 18 mM, rather than 20 mM, Tris-HCl pH 7.5 during the final size exclusion step (Bensing et al., 2022).

*sTa-(N-Fmoc)Ser synthesis.* Chemoenzymatic synthesis of sTa-( *N* -Fmoc)Ser was performed as described, reported as compound **25** (Lau et al., 2011).

*Crystallography.* SLBR _GspB–Siglec_ (22.4 mg/ml) was pre-incubated with 10 mM sTa-( *N* -Fmoc)Ser, then crystallized and cryo cooled as described (Bensing et al., 2022). Diffraction data were collected using a wavelength of 0.97951 Å at ESRF beamline BM7 on a Pilatus 6M detector. Data were processed using the HKL suite (Otwinowski & Minor, 1997). The structure was determined by molecular replacement in PHENIX using PDB 5IUC as the search model (Adams et al., 2010; Pyburn et al., 2011). The model was improved in COOT (Emsley et al., 2010), and refinement was performed in PHENIX (Adams et al., 2010).

*Data availability.* Raw diffraction data are deposited in SBGRID under accession code 1193. Coordinates and structure factors are deposited in the PDB under accession code 9Q3H. pBG101-SLBR _GspB-Siglec_ is available by request.

## Reagents

**Table:** 

*E. coli* BL21 (DE3)	T7 expression strain	Agilent Technologies
pBG101-SLBR _GspB-Siglec_	pBG101 is a pET27 derivative containing an N-terminal His _6_ -GST tag, subcloned from pGEX, followed by a 3C precision protease site. pBG101-SLBR _GspB-Siglec_ contains residues 398 – 521 of full-length GspB	pET27 and pGEX from Sigma Aldrich. pBG101 created by Vanderbilt University Center for Structural Biology
sTa-( *N* -Fmoc)Ser	Neu5Acα2,3-Galβ1,3-GalNAcα-serine-9-fluorenylmethyloxycarbonyl	Multienzyme synthesis, described as compound 25 in Lau, 2011.
All other chemicals		Sigma Aldrich
